# Older adults in phase I clinical trials: a comparative analysis of participation and clinical benefit rate among older adults versus middle age and AYA patients on phase I clinical trials with VEGF/VEGFR inhibitors

**DOI:** 10.18632/oncotarget.25571

**Published:** 2018-06-22

**Authors:** Ishwaria M. Subbiah, Chad Tang, Arvind Rao, Gerald S. Falchook, Vivek Subbiah, Apostolia M. Tsimberidou, Daniel Karp, Razelle Kurzrock, David S. Hong

**Affiliations:** ^1^ Department of Palliative, Rehabilitation and Integrative Medicine, University of Texas MD Anderson Cancer Center, Houston, TX, USA; ^2^ Division of Radiation Oncology, University of Texas MD Anderson Cancer Center, Houston, TX, USA; ^3^ Department of Bioinformatics and Computational Biology, University of Texas MD Anderson Cancer Center, Houston, TX, USA; ^4^ Sarah Cannon Research Institute at HealthONE, Denver, CO, USA; ^5^ Department of Investigational Cancer Therapeutics (Phase I Program), University of Texas MD Anderson Cancer Center, Houston, TX, USA; ^6^ Center for Personalized Cancer Therapy and Clinical Trials Office, Moores Cancer Center, University of California, San Diego, CA, USA

**Keywords:** geriatric oncology, elderly, VEGF inhibitors, older adults, phase I trials

## Abstract

**Background:**

Older adults aged 65 years and above remain underrepresented in cancer clinical trials. We hypothesized that older participation in early phase trials with VEGF/VEGFR (VEGF/R) inhibitors was lower than cancer prevalence in this group and lower than other age groups (middle age, adolescent/young adults [AYA]).

**Results:**

Of 1489 patients, 278 were older adults (18%, median age 68.9y), 220 AYA (15%, median age 32.6 y), 991 middle age (67%, median age 53.8 y). Common malignancies included gastrointestinal (*n* = 438, 29%), gynecologic (*n* = 234, 16%), and thoracic/head/neck (*n* = 216, 15%). Median time to treatment failure did not vary significantly between the 3 age-based cohorts (3m in older adults, 3.5 m middle age, 3.3 m AYA). OR of achieving clinical benefit in older adults vs middle age (OR 1.10, p 0.19 [two-tailed], p 0.09 [one-tailed]) and AYA vs middle age (OR 0.85, p 0.31 [proportions *z*-test, two tailed], p 0.15 [one-tailed]) showed no significant differences.

**Conclusions:**

Older adults accounted for <20% of participants on phase I clinical trials with VEGF/R inhibitors but those who participated were just as likely to achieve a clinical benefit as AYA and middle age patients. These findings merit further exploration into patient selection for early phase trials.

**Methods:**

We identified and separated patients treated on VEGF/R-inhibitor-based phase I trials from 12/1/2004–07/31/2013 into 3 age-based cohorts, AYA (15–39y), middle age (40–64 y), older adults (65 y+). We analyzed clinical/treatment characteristics and response outcomes, calculating the odds ratios (OR) of clinical benefit (defined as SD ≥ 6months, PR, CR) for older adults and AYAs versus middle age participants.

## INTRODUCTION

Tumor angiogenesis and its associated role in cancer growth and metastases have long been identified as therapeutic targets in the management of cancer [1, 2]. Agents targeting vascular endothelial growth factor family and their receptors (VEGF/R), including anti-VEGFA monoclonal antibodies and a host of receptor tyrosine kinase inhibitors, have been approved by the US Food and Drug Administration (FDA) and the European Medicines Agency (EMEA), first starting with bevacizumab in combination with an intravenous 5-fluorouracil (5-FU)-based chemotherapy regimen for metastatic colorectal cancer [3, 4]. The approved indications include the treatment of cancers of the gastrointestinal tract, hepatobiliary system, renal cell carcinoma, thyroid cancers, and soft tissue sarcoma [5]. Furthermore the drug development pipeline is robustly populated by novel agents with VEGF/R inhibition, particularly as novel combinations with immunotherapies [6].

Given this prominent presence of antiangiogenic agents in the anticancer armamentarium, the characteristics of patients participating on the early phase clinical trials of these agents becomes of particular concern [7]. Specifically, precedent dictates that the age distribution of patients participating on cancer clinical trials overall does not reflect the age distribution of the general population with advanced cancer, both in incidence and prevalence. [8, 9]. Indeed despite accounting for a majority of new cases of cancer as well as cancer-related mortality in the United States, older adults aged 65 and above have been consistently underrepresented in oncology clinical trials [10, 11]. This age-based discrepancy in participation of older adults is more so evident in early phase I/II clinical trials, particularly with novel agents [12]. Indeed the heterogeneity among trials participants in early phase trials becomes of particularly high significance given that these trials are meant to identify therapy-associated toxicities of all severities and preliminary insight into efficacy.

To that end, we hypothesized that older adults aged 65 years and above remain underrepresented on phase I trials with drugs targeting VEGF/R. Furthermore, we hypothesized that older adults who do participate on these phase I trials have a comparable probability of achieving clinical benefit when compared to middle age patients and adolescent/young adult (AYA) patients.

## RESULTS

### Patient characteristics

Overall, we identified 1,489 consecutive patients who were treated on a phase I clinical trial with drugs that have a component of VEGF/R inhibition (Table 1). Of these patients, 278 (18%) were older adults aged 65 years and above (median age 68.9y, range 65 to 86.3 years), 991 (67%) were middle age (median age 53.8 years, range 40 to 64.9 years), and 220 (15%) were adolescent and young adults (AYA, median age 32.6 years, range 15.4 to 39.9 years). There were no significant differences in gender among the older adults and middle age cohorts, with women representing 46% of older adult patients treated on trial and 55% of middle age patients. However among the AYA subgroup, the gender gap was more evident with women comprising a larger percentage of trial participants than men (67% women, 33% men).

**Table 1:** Characteristics of the 1,489 patients treated on an anti-VEGF/R-based phase I trial

	AYA (15–39 y)	Middle age (40–64 y)	Older adults (65+y)
*n*	220 (15%)	991 (67%)	278 (18%)
Age, median, years	32.6	53.8	68.9
Age, range, years	15.4–39.9	40.0–64.9	65.0–86.3
Gender			
Female	148 (67%)	548 (55%)	127 (46%)
Male	72 (33%)	443 (45%)	151 (54%)
#prior therapies, median (range)	3 (0–15)	3 (0–13)	3 (0–13)
Primary cancer			
Breast	20 (9%)	95 (10%)	14 (5%)
Endocrine	17 (8%)	60 (6%)	13 (5%)
Gastrointestinal	35 (16%)	306 (31%)	97 (35%)
Genitourinary	7 (3%)	81 (8%)	20 (7%)
Gynecologic	41 (19%)	160 (16%)	34 (12%)
Melanoma	25 (11%)	90 (9%)	30 (11%)
Other	5 (2%)	20 (2%)	17 (6%)
Sarcoma	39 (18%)	46 (5%)	10 (4%)
Thoracic/Head/Neck	31 (14%)	133 (13%)	43 (15%)

All trial participants were heavily pretreated with a median number of prior therapies being 3 in all three-age based cohorts. Overall, among the 1,489 patients, the most common malignancies were gastrointestinal (*n* = 438, 29%), gynecologic (*n* = 234, 16%), and thoracic/head/neck (*n* = 216, 15%). Gastrointestinal tumors were the most common primary malignancy among the middle age (31%, *n* = 306) and older adults (35%, *n* = 97). However the gynecologic cancers (19%, *n* = 41) and sarcomas (18%, *n* = 39) were more common than gastrointestinal primaries (16%, *n* = 35) among the AYA subgroup in comparison to the middle age and older adults.

### Treatment characteristics

Overall, 472 of the 1,489 patients (32%) were treated on protocol with single agents, while 1017 (68%) were given a combination therapy with 2 or more agents (Table 2). Specifically, 648 (44%) patients received two agents, 362 (24%) received three, and 7 (<1%) received four. Among the 278 older adults, 98 (35%) received monotherapy compared to 298 (30%) of 991 middle age patients and 76 (35%) of 220 AYA patients. Of the 278 older adults, 180 (65%) received combination therapy with either two (47%, *n* = 132), three (17%, *n* = 46), or four (<1%, *n* = 2) drugs. Overall, 575 (69%) of the 1489 patients received a novel agent that, at that time, was not FDA-approved for any indication, including 110 (40%) of the older adults, 369 (37%) of the middle age participants, and 96 (44%) of the AYAs; those drugs that were FDA-approved were under trial here in a dose-finding study for new indications.

**Table 2:** Treatment characteristics of the three age-based cohorts

	AYA (15–39 y)	Middle age (40–64 y)	Older Adults (65+y)	Total
Total number of patients	220	991	278	1489
Monotherapy	76 (35%)	298 (30%)	98 (35%)	479 (32%)
Combination therapy	144	693	180 (65%)	1017 (68%)
2 drugs	90	426	132 (47%)	648 (44%)
3 drugs	54	262	46 (17%)	362 (24%)
4 drugs	0	5	2 (<1%)	7 (<1%)
Drug(s) given on protocol				
Non-FDA approved agent	96 (44%)	369 (37%)	110 (40%)	575 (69%)
Bevacizumab	76	340	80	496 (33%)
TKI	45	204	58	307 (21%)
Sorafenib	20	87	21	128 (42%)
Cabozantinib	7	51	15	73 (24%)

Abbreviations: FDA Food and Drug Administration.

Overall, 1489 patients enrolled on 106 phase I trials that included at least one agent with anti-angiogenic activity and were consequently included in this analysis. All 106 trials enrolled patients with all advanced cancers, 75 (71%) were industry-sponsored while 31 (29%) were investigator-initiated studies. The studies themselves did not have an upper age limit but approximately 30% did exclude patients aged below 15 years. All trials were intended to obtain the maximum tolerated dose of the drug (or at least one of the drugs in a combination study) – this agent with the variable dosing was not universally the anti-angiogenic drug in a multi-drug combination phase I trial.

The monoclonal antibody bevacizumab (33%, *n* = 496) was the most common type of anti-angiogenic agent given in our 1489 trial participants with tyrosine kinase inhibitors (TKI) being the second (21%, *n* = 307). Of these 307 patients, the most common TKIs administered that had anti-angiogenic properties were sorafenib, given to 42% (*n* = 128) of patients. Also frequently used were cabozantinib (24%, *n* = 73), pazopanib (10%, *n* = 32) and a novel TKI targeting the VEGFR/PDGFR kinase family (9%, *n* = 28).

### Time to treatment failure (TTF)

To recap, the overall time to treatment failure was defined as the time in months between the first day of the first cycle to the date off study due to clinical and/or radiographic progression or date of death if that preceded the anticipated date of restaging imaging. When comparing the time on study of the three age-based cohorts, the median time to treatment failure on a phase I trial did not vary significantly between the 3 cohorts with the older adults being 3 months, the middle age patients being 3.5 months, and the AYAs being 3.3 months.

### Response on trial

Over the 1489 patients treated on a phase I trial, 729 (49%) attained a stable disease (SD) per RECIST as their best response on trial, of whom 306 (21%) patients reached a prolonged SD of 6 months or longer (median duration 9.2 months; Table 3). Additional responders included 158 (11%) patients who attained a partial response (PR) with a median duration of response being 9.6 months and 8 (<1%) patients with a complete response (CR, median duration of response 37.4 months). Among the 278 older adults, 136 (49%) attained SD including 53 (19%) patients with prolonged SD of 6 months or more (median duration of response 9.0 months) and 23 (8%) attained a PR (median duration 7.4 months). Among middle age patients, 500 (50%) had an SD including 211 (21%) prolonged SD (median duration 9.2 months), 101 (10%) patients with a PR (median duration 10.8 months) and 6 (<1%) patients with a CR (median duration 29.3 months). In the 220-patient AYA cohort, 93 (42%) attained SD with 42 (19%) prolonged SD (median duration 9.7 months), 34 (15%) had a PR (median duration 9.5 months), and 2 patients had a CR lasting 36.2 months and 50.1 months. Overall, 78 (35%) AYA participants, 384 (32%) middle age patients and 119 (27%) older adults achieved a clinical benefit (CR + PR + SD > 6 months).

**Table 3:** Clinical responses per RECIST and clinical benefit rate based on age

	AYA (15–39 y)	Middle age (40–64 y)	Older Adults (65+y)	Total
Total number of patients	220	991	278	1489
Complete response (CR)	2 (1%)	6 (1%)	0 (0%)	8 (<1%)
Partial response (PR)	34 (16%)	101 (10%)	23 (8%)	158 (11%)
Stable disease (SD) <6 months	51 (23%)	289 (29%)	83 (30%)	423 (28%)
Prolonged SD >6 months	42 (19%)	211 (21%)	53 (19%)	306 (21%)
Progressive disease	91 (41%)	384 (39%)	119 (43%)	594 (40%)
Clinical benefit rate (CBR)	78 (35%)	318 (32%)	76 (27%)	472 (32%)

Abbreviations: AYA Adolescent and young adults. CBR Clinical benefit rate ([CR + PR + SD > 6 m]/number of patients in that age cohort).

### Odds ratio of achieving clinical benefit

We then analyzed the likelihood of achieving a clinical benefit among the three age-based cohorts while on study. The odds ratio of achieving clinical benefit in older adults in comparison to middle age patients was 1.10, p 0.19 (two-tailed), p 0.09 (one-tailed). Similarly, the odds ratio of achieving clinical benefit in among the AYA patients versus middle age patients is 0.85, p 0.31 (proportions *z*-test, two tailed), p 0.15 (one-tailed). No significant differences were found in the odds ratio or likelihood of attaining a clinically beneficial response between the older adults, AYA, and middle age cohorts.

## DISCUSSION

Overall in our study, patients aged 65 years and above accounted for less than 20% of all participants on phase I clinical trials with VEGF/R inhibitors but those older adults who did participated were just as likely to achieve a clinical benefit of disease response as the other age-based cohorts (AYA and middle age patients). For older adults, the oncologic decision-making process takes on additional complexities in the context of age-related changes including decline in organ function, comorbidities as well as the surrogate assessments of frailty such as performance status as determined by the medical oncologist [15, 16]. Retrospectively, pooled analysis from randomized phase III studies of bevacizumab in metastatic colorectal cancer has shown that older adults who met the trial inclusion criteria assessing their medical fitness for participation had comparable progression-free survival and overall survival as the patients who were aged 65 and younger [11, 17]. The conscious identification of enrollment disparities of population subgroups such as older adults and women has led to a trend in recent years towards a greater representation of these groups in cancer clinical trials [18]. Prospectively, such improvements in older adult participation have been particularly recognized among non-small cell lung cancer patients participating in the National Cancer Institute cooperative groups trials, where the enrollment disparity of the older adults showed a considerable improvement over a 12-year period from 1990 to 2012 [19].

However, our findings suggest that such increases in participation may not have yet extended to early phase studies. With the advent of personalized oncology practice and the movement to tailor therapies to the individual, patients including older adults may have identified tumor-associated aberrations against which non-approved agents are available on clinical trials [20, 21]. However, this access to early phase trials by all subpopulations including older adults begins with the identification of barriers to clinical trial enrollment [22, 23]. Phase I/II trials are generally viewed as more experimental therapies of non-FDA-approved drugs with much less clinical information (sometimes, no substantial clinical data in first-in-human studies) on tolerance, toxicities, and certainly efficacy [24, 25]. Beyond the trial-mandated inclusion criteria and determination of comorbidities, organ function and performance status (using the ECOG or Karnofsky scales), the subjective perception of frailty in an older patient by the treating oncologist and research team in the clinic can manifest as a lower likelihood to consider or offer early phase trials for older adults [26].

In our study, we hope to add to the body of data on the performance of older adults on clinical trials, in this case on early phase I trials with VEGF/R targeting agents. In our experience, older patients accounted for less than 20% of patients on these phase I trials but those who participated were just as likely to achieve a clinical benefit as the adolescent and young adult patient or the middle age patients. This outcome brings to light the limitations of this study. Above all, the selection bias exists where presumably only the most ‘medically fit’ older adults ultimately received therapy on trial whereas consideration must be given to how many older adults seen in the clinic were informally not considered for the trials and how many did not progress through the formal screening process for each trial. Additionally, analyses are underway to thoroughly characterize the treatment-related toxicities and their association with clinical outcomes to be reported in the near future. Data on distribution of the older adults across dose levels within each protocol as well as the half maximal inhibitory concentration (IC50) of the select anti-VEGF/VEGFR agents that were not commercially available was not readily accessible for our review; however earlier work has demonstrated that the outcomes including time to treatment failure, response rate, progression free survival and overall survival were not significantly different between the patient treated on lower dose levels where the treatment dose was 25% or less of the maximum tolerated dose (MTD) versus high dose level where the treatment dose was 75% or higher of the MTD [27]. Finally, the information gathered during our data collection process highlighted the absence of universal adoption of geriatrics-specific assessment including assessments for frailty, polypharmacy, etc. We emphasize the importance of a thorough geriatric assessment of patients aged 65 and above as a part of their oncologic care particularly in the advanced cancer setting under consideration for novel therapeutics.

Indeed, Identification of clinician and patient-specific determinants to decision making on participation in an early phase trial will be crucial to improving the participation of older adults on phase I trials and removing this enrollment disparity [25]. The emergence of geriatric oncology and the emphasis on improved access for older adults to newly approved cancer therapies as well as clinical trials highlights the importance to independently study the evolution of participation of patients in the special age groups, i.e. older adults, adolescent and young adults, over the past one to two decades, an area of future study. The results of early phase clinical trials and subsequent trials of efficacy not only shape national clinical guidelines for cancer treatment but also factor heavily into patient-doctor therapeutic decision-making. Therefore having the appropriate heterogeneity among clinical trial participants to reflect the actual patient population likely to receive this therapy in the general community remains a responsibility for clinician investigators.

## PATIENTS AND METHODS

### Patient characteristics

We queried a prospectively maintained departmental database of patients evaluated in the Department of Investigational Cancer Therapeutics between December 1, 2004 and July 31, 2013. We identified consecutive patients with advanced solid tumors who met inclusion criteria for and consequently treated on phase I clinical trials with VEGF/R inhibitors to be included in this analysis. We then separated them into 3 age-based cohorts: AYA (15–39 years), middle age (40–64 years), and older adults (65 years and above) as defined by the National Comprehensive Cancer Network (NCCN). Clinical information collected included gender, date of birth, tumor type, date of diagnosis, Eastern Cooperative Oncology Group (ECOG) performance status at start of phase I trial, start date (cycle 1, day 1) of the phase I trial, and age on the trial's start date. This study and associated clinical trials were conducted in accordance with the guidelines of the MD Anderson Institutional Review Board and patient confidentiality was maintained following Health Insurance Portability and Accountability Act guidelines.

### Treatment and evaluation

Patients included in the analysis met trial-specific inclusion criteria and received therapy on that phase I trial. Patients received care such as laboratory testing and interim clinician visits as specified by each protocol. Elements of care standard throughout the protocols were as follows. Patients underwent a baseline clinical exam with the medical oncologist along with subsequent follow-ups prior to each cycle and as clinically warranted. Imaging (either CT, MRI, MRI PET/CT as specific by protocol) was obtained at baseline and prior to alternating treatment cycles usually at 6–8 week intervals. Imaging response was assessed using Response Evaluation Criteria In Solid Tumors (RECIST) criteria [13, 14]. Patient remained on therapy until disease progression (radiologic progression on restaging imaging or clinical progression where the patient's overall performance status declined before restaging imaging could be obtained), unacceptable toxicity not manageable with optimum medical therapy, patient death or withdrawal of consent. The date of death was obtained from the electronic medical record; patients lost to follow up were censored at the date of last follow-up.

### Statistical analysis

Categorical variables are listed in the tables and descriptive statistics were employed to analyze continuous variables. The clinical benefit, defined as a response of stable disease (SD) ≥6 months, partial response (PR), or complete response (CR), per RECIST, was determined for each age-based cohort. We developed a contingency table analysis (two-tailed Fisher's exact test) to examine the association between pairs of categorical variables, the three age cohorts and likelihood of achieving a clinical benefit; we calculated the odds ratios (OR) of achieving a favorable clinical benefit for the 3 age cohorts, and for older adults and AYA in comparison to the middle age cohort. Statistical analyses were carried out using statistical software (The R Foundation for Statistical Computing, version 2.15, Vienna University of Economics and Business, Vienna, Austria).
